# Evaluation of Ethanolic Powdered Extract of *Magnolia tamaulipana* Vazquez against *Oligonychus punicae* Hirst (Trombidiformes: Tetranychidae)

**DOI:** 10.3390/plants11131711

**Published:** 2022-06-28

**Authors:** Francisco Reyes-Zepeda, Rapucel Tonantzin Quetzalli Heinz-Castro, Fabian Eliseo Olazaran-Santibañez, Salvador Ordaz-Silva, José Guadalupe Pedro-Méndez, Julio César Chacón-Hernández

**Affiliations:** 1 Institute of Applied Ecology, Universidad Autónoma de Tamaulipas, Ciudad Victoria 87019, Mexico; freyes@docentes.uat.edu.mx (F.R.-Z.); feolazaran@docentes.uat.edu.mx (F.E.O.-S.); 2 Faculty of Agronomy and Veterinary, Universidad Autónoma de San Luis Potosí, Soledad de Graciano Sánchez 78321, Mexico; rapucel.heinz@uaslp.mx; 3 Faculty of Business and Engineering San Quintín, Universidad Autónoma de Baja California, San Quintín 22930, Mexico; salvador.ordaz.silva@uabc.edu.mx (S.O.-S.); jpedro@uabc.edu.mx (J.G.P.-M.)

**Keywords:** avocado bronze mite, antioviposition, antifeeding, biological control, integrated pest management

## Abstract

Avocado bronze mite (ABM), *Oligonychus punicae* Hirst (Trombidiformes: Tetranychidae) has potential for development in several plant species of agricultural importance. ABM is one of the most economically important pests in avocado cultivars, causing major damage to fruit and defoliation. At present, the control of ABM depends mainly on agrochemicals. Therefore, it is necessary to find alternatives to agrochemicals that can help minimize environmental impact and health risks for humans and mammals. The aim of this research was to assess the effect of different concentrations (5, 10, 50, 100, 250, 500, 1000 µg/mL) of ethanolic powdered extract of *M. tamaulipana* leaves against adult ABM females. The different concentrations of *M. tamaulipana* extract did not cause mortality of *O. punicae*. Females treated with 5 and 1000 µg/mL of the extract showed a decrease in the number of eggs laid per female at 24 (5.17 and 1.27), 48 (5.07 and 1.17), and 72 h (4.97 and 0.80), compared to the control treatment (5.20, 6.60 and 6.87), respectively, which led to a reduction in the growth rate. Percentage of feeding damage decreased with the increasing concentration of the extract. The ethanolic powdered extract of *M. tamaulipana* leaf has potential to control *O. punicae*.

## 1. Introduction

Avocado bronze mite (ABM), *Oligonychus punicae* Hirst (Trombidiformes: Tetranychidae) is a major pest of avocado (*Persea americana* L.) crops in Southern California (McMurtry and Johnson 1966) and Mexico [1,2]. ABM feeds primarily on the upper leaf surface, but in high populations, this mite moves to the lower leaf surface to feed. ABM inserts its stylet into the leaf tissue, sucking out the cell contents, and the cell remnant coagulates to form a yellowish to brown necrotic mass [3]. High populations of these mites cause defoliation, leading to sunscalds in fruits and causing fruit drops due to fruit-setting abortion, providing lower yields. *O. punicae* is controlled through the use of agrochemicals [1]. However, ABM develops resistance to chemical insecticides very quickly, due to its short life cycle and high fecundity and fertility [1,4,5,6,7]. Therefore, research on alternatives for the management of *O. punicae* is required.

Plant-derived extracts are an alternative method to chemicals insecticides and, in addition, offer an ecological solution with a minimal detrimental effect on the environment, humans, and mammals [8,9,10]. Some species of *Magnolia*, such as *M. citrata*, *M. dealbata*, *M. fargesii*, *M. kobus*, *M. schiedeana*, and *M. tamaulipana*, have a diversity of secondary metabolite compounds with insecticidal and acaricidal properties [11,12,13,14,15,16]. *M. tamaulipana* trees grow in the cloud forest and are endemic to UNESCO Biosphere Reserve, El Cielo, located in Tamaulipas, Mexico [17,18]. Literature contains reports stating that *M. tamaulipana* leaves have several secondary metabolites such as flavonoids, tannins, sterols and terpenes, saponins, carotenoids, carbohydrates, purines, free reducing sugars, soluble starch, and quinones [19]. A few researchers have reported that *M. tamaulipana* has spider mite control properties. The ethanolic powdered extract from *M. tamaulipana* leaf has anti-oviposition activity and anti-feeding properties on *Tetranychus urticae* Koch [15]. The aim of this research was to assess the effect of different concentrations (5, 10, 50, 100, 250, 500, 1000 µg/mL) of ethanolic powdered extract of *M. tamaulipana* leaves against adult ABM females.

## 2. Results

### 2.1. Phytochemical Composition

The ethanolic extract of *M. tamaulipana* leaves has several groups of secondary metabolites, such as flavonoids, tannins, sterols and terpenes, saponins, carotenoids, carbohydrates, purines, free reducing sugars, soluble starch, and quinones. Alkaloids did not appear in the extract (Table 1).

### 2.2. Oviposition

**Figure 1:**
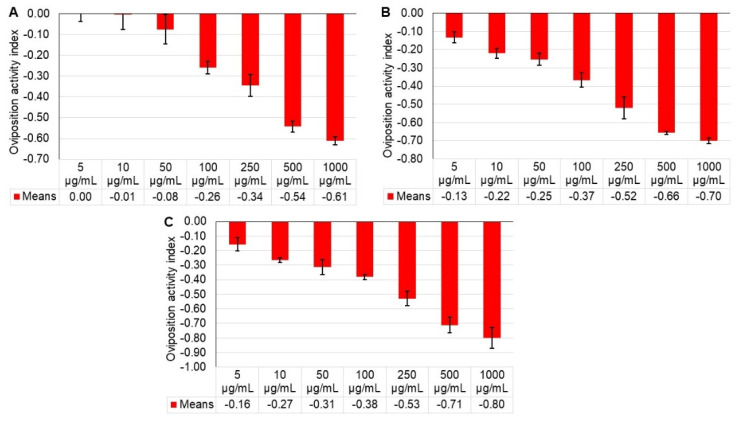
Ovipositional activity index (mean ± standard error) for *Oligonychus punicae* in response to different concentrations of *Magnolia tamaulipana* ethanolic powdered extract at (**A**) 24 h, (**B**) 48 h, and (**C**) 72 h.

We evaluated the effect of seven concentrations of ethanolic extract of *M. tamaulipana* leaves against adult females of ABM. The extract did not cause mortality in the *O. punicae* females, but did cause a reduction in the number of laid eggs and food intake of ABM. At 24, 48, and 72 h after extract application, there was a significant effect on the egg-laying ability of ABM females (F = 44.80; df = 7, 16; *p* < 0.0001; F = 84.55; df = 7, 16; *p* < 0.0001; F = 47.13; df = 7, 16; *p* < 0.0001, respectively (Table 2)). At 24 h, the oviposition activity index ranged between 0.0014 (5 µ/mL) and 0.6102 (1000 µ/mL), at 48 h, it was between 0.1324 (5 µ/mL) and 0.6997 (1000 µ/mL), and at 72 h it was between 0.1564 (5 µ/mL) and 0.7993 (1000 µ/mL), as compared to the control treatment (Figure 1). The effective concentration EC_50(90)_ of 308.16 (2982) µg/mL resulted in 50% fewer eggs laid compared to the control. The results showed that the oviposition of *O. punicae* females decreased as the extract concentration increased.

### 2.3. Feeding Damage

**Figure 2:**
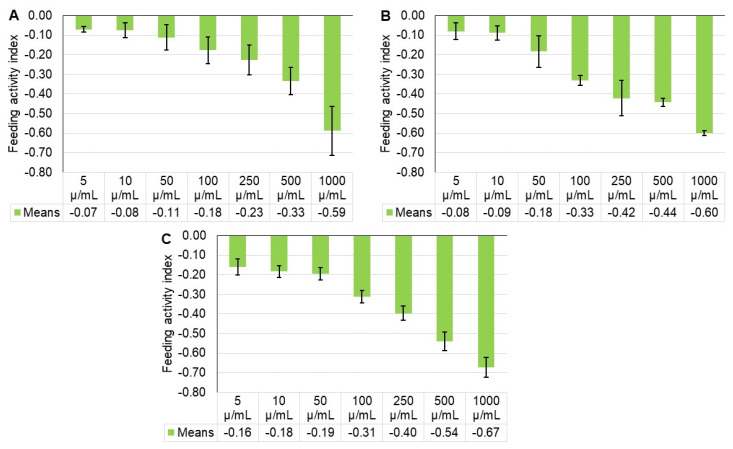
Feeding activity index (mean ± standard error) for *Oligonychus punicae* in response to different concentrations of *Magnolia tamaulipana* ethanolic powdered extract at (**A**) 24 h, (**B**) 48 h, and (**C**) 72 h.

The damage percentage differed significantly among the treatments, at 24, 48, and 72 h (F = 12.86; df = 7, 16; *p* < 0.0001; F = 18.59; df = 7, 16; *p* < 0.0001; F = 40.08; df = 7, 16; *p* < 0.0001), respectively (Table 3). Feeding activity of ABM females treated with 5 to 1000 µ/mL of *M. tamaulipana* extract was inhibited by 0.071 to 0.587, 0.080 to 0.599, and 0.160 to 0.673 at 24, 48, and 72 h, respectively, as compared to the control treatment (Figure 2). The results showed that the feeding damage of *O. punicae* females decreased as the extract concentration increased.

**Figure 3:**
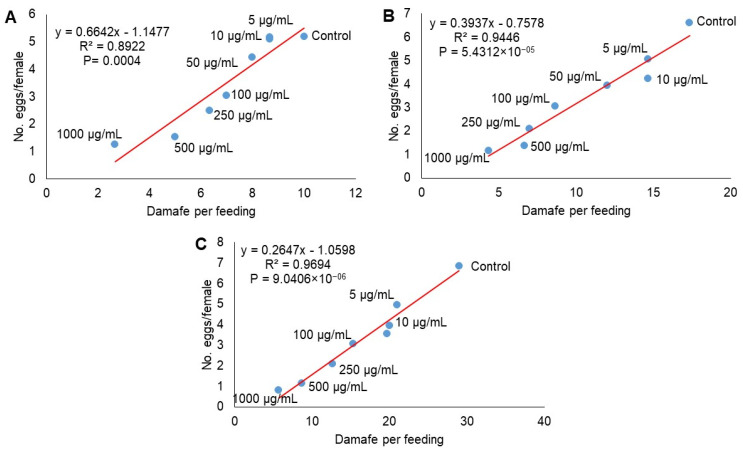
The correlation between the number of eggs laid per female and percentage of feeding damage of *Oligonychus punicae* treated with different concentrations of ethanolic powdered extract of *Magnolia tamaulipana* leaves at 24 h (**A**), 48 h (**B**), and 72 h (**C**).

The oviposition rate and the percentage of feeding damage of *O. punicae* decreased as concentration rates increased (Figure 3). The regression method showed that the number of eggs per female depended on the feeding of ABM females that were treated with different concentrations of *M. tamulipana* extract. The values of R^2^ indicated that 89.22% (F = 49.67; df = 1, 6; *p* = 0.0004), 94.46% (F = 102.28; df = 1, 6; *p* = 5.4312 × 10^−5^) and 96.94% (F = 190.20; df = 1, 6; *p* = 9.0406 × 10^−6^) of the variation in the number of eggs laid per *O. punicae* female was associated with feeding damage at 24, 48, and 72 h, respectively. The results showed that the increase in the concentration of the extract of *M. tamaulipana* negatively influences the relationship between the oviposition and feeding damage of ABM females.

### 2.4. Demographic Parameters

The growth rate of *O. punicae* was significantly different among the treatments (F = 315.51; df = 7, 16; *p* < 0.0001) and it decreased as the concentration increased (Table 4). The highest average (±SE) of the r of *O. punicae* was observed in the control group (0.9929 ± 0.01) and the lowest r was in females treated with 1000 µg/mL (0.4809 ± 0.01). The number of mites added to the population per female per day (λ) was significantly greater in the control group than in mites treated with the extract (F = 367.61; df = 7, 16; *p* < 0.0001). The doubling time (DT) of ABM differed significantly between the treatments (F = 170.38; df = 7, 16; *p* < 0.0001). The average time in which the ABM population doubled was greater when females were treated with 1000 µg/mL of the extract (1.4421 ± 0.02) and less pronounced in the control group (0.6982 ± 0.00) (Table 4).

## 3. Discussion

As in Arredondo-Valdés et al. [19], in this work, we found that *M. tamaulipana* leaf ethanolic powdered extract has different secondary metabolites, including flavonoids, tannins, sterols and terpenes, saponins, carotenoids, carbohydrates, purines, free reducing sugars, soluble starch, and quinones. Secondary plant metabolites such as alkaloids, saponins, phenols, and terpenes can be useful to pest management strategies. These phytochemicals affect oviposition and intake feed, as well as retarding growth, causing chemo-sterilization, and leading to the death of several herbivorous arthropod species [20,21].

Oviposition of phytophagous arthropods over their host plants often precedes herbivory [22]. Therefore, controlling the oviposition of *O. punicae* female is essential because, over time, the population of the next generation of ABM is reduced. This research shows that the *O. punicae* inhibition rate increases in relation to higher concentrations of the *M. tamaulipana* extract. Our results are similar to those reported by Chacón-Hernández et al. [15]. They reported oviposition inhibition rates of *T. urticae* at concentrations of 5, 10, 50, 100, 250, 500, and 1000 µg/mL of *M. tamaulipana* ethanolic powdered extract. The oviposition inhibition rate increased at 24 h (18.18% to 95.56%), 48 h (7.69% to 95.83%), and 72 h (11.74% to 95.39%), as compared to the control. On the other hand, the effects of other plant extracts on the tetranychids’ oviposition have been studied and those results are similar to the results of this work. Sivira et al. [23] documented that ethanolic extracts of *Lippia origanoides* H.B.K. (Verbenaceae) and *Gliricidia sepium* (Jacq.) Kunth ex Walp. (Fabaceae) reduced the oviposition of *T. cinnabarinus* Boisduval at 72 h (43.7% and 57.0%) at a concentration of 5% (*v*/*v*), as compared to the control. Pavela et al. [24] found that the concentrations (0.5, 1.0, 5.0, 10.0, 15.0, 20.0, and 30.0 g·L^−1^) of *Saponaria officinalis* L. (Caryophyllaceae) root crude extract inhibited oviposition of *T. urticae* females (3.1% to 94.1%), as compared to the control at 48 h. Pavela et al. [25] reported that different concentrations (25, 50, 80, 100, and 150 g cm^−2^) of methanolic and ethyl acetate extracts from *Tithonia diversifolia* (Hemsl.) A.Gray (Asteraceae) inhibited the egg-laying capacity of *T. urticae*, ranging between 9.8 to 62.8% and 26.9 to 100.0%, respectively, in contrast with the control. In addition, Ribeiro et al. [26] documented that ethanolic extract of *Annona mucosa* Jacq. (Annonaceae) seeds reduces the egg-laying activity (1.70 eggs/female) of *Panonychus citri* (McGregor) (Acari: Tetranychidae) at a concentration of 10,000 mg L^−1^ in 5 days, as compared to the control (23.29 eggs/female). The anti-oviposition of ABM could be due to presence of flavonoids in the *M. tamaulipana* ethanolic powdered extract (Table 1). In this regard, Vásquez et al. [5] found a negative relationship between flavonoid content of grape cultivars and *O. punicae* oviposition. Dimetry et al. [27,28] mentioned that mites’ anti-oviposition may be due to two situations. First, secondary plant metabolites can directly influence the female ovaries. Second, by direct contact of mite’s cuticle with one or more bio-active compounds present in the botanical extract can alter the production of pheromones. On the other hand, Hosny et al. [29] mentioned that both chemical and botanical products with acaricidal properties can partially or temporarily sterilize the females, which causes a lower number of eggs laid/female/day, as well as a lower number of hatched eggs, as compared to the control group. According to the previous information, although more research is required, the ethanolic powdered extract of *M. tamaulipana* leaves causes anti-oviposition and can be characterized by its sterilizing effect.

Feeding damage decreased as the concentration rates increased. Similarly, Chacón-Hernández et al. [15] found that different concentrations (5, 10, 50, 100, 250, 500, and 1000 µg/mL) of *M. tamaulipana* ethanolic powdered extract have anti-oviposition effects on *T. urticae* females. They reported an oviposition inhibition rate increase between 18.18% to 95.56%, 7.69% to 95.83%, and 11.74% to 95.39%, according to the concentration of the extract, at 24, 48, and 72 h, respectively. Fetoh and Al-Shammery [30] documented that the lethal concentration of 50% (LC_50_, 47.6, 1102, and 8433.2 ppm) of the ethanolic extracts from *Cuminum cyminum* (Labiaceae), *Duranta plumeria* (Verbenaceae) and *Ambrosia maritimal* (Compositae) deter feeding of *Oligonychus afrasiaticus* McGregor on bean plants (*P. vulgaris*) by 95.33% to 97.80%, 66.67% to 97.80%, and 53.33% to 93.33%, at 24, 48, and 72 h, respectively. The anti-feeding of *O. punicae* can be due to presence of terpenes and flavonoids in the *M. tamaulipana* ethanolic powdered extract (Table 1). In this regard, Mierziak et al. [31] and Koul [32] mentioned that secondary metabolites such as alkaloids, flavonoids, and terpenes are the most potent antifeedants. Therefore, after applying the extract of *M. tamaulipana* directly to *O. punicae* females, possibly some secondary metabolite entered into contact with its gustatory and olfactory sensory systems so that its palatability was altered, reducing its food intake.

## 4. Materials and Methods

### 4.1. Colonia de Red Spider Mite Colony

A *O. punicae* colony was started with biological material obtained from the Population Ecology Laboratory, Institute of Applied Ecology, Autonomous University of Tamaulipas. To increase the avocado bronze mite population, female and male *O. punicae* were placed on bean plants (*Phaseolus vulgaris* L. (Fabaceae)) under greenhouse conditions at 29 ± 4 °C and 60 ± 15% relative humidity (RH).

### 4.2. Preparation of the Plant Material and the Extract

Uncontaminated leaves of *M. tamaulipana* (i.e., without external agents and physical contaminants) were exposed to sunlight to remove their moisture [33]. The dry leaf samples were ground into a fine powder by an electric mill (Cuisinart DBM-8, Stamford, CT, USA). The powder of *M. tamaulipana* (150 g) was mixed with 96% ethanol (500 mL) to prepare the extract that was kept under constant stirring on Thermo Scientific™ Cimarec™ Digital Stirring Hotplates for three days at room temperature (27 °C), in amber bottles covered with aluminum [34]. Subsequently, the solution was filtered using Whatman No. 4 paper. The solvent was removed from the extract by passing it through a vacuum system on a rotary evaporator at 60 °C for two hours (Yamato Scientific America Inc., Model-RE301, Santa Clara, CA, USA), and the rest of the solvent was dried in an oven chamber (Shel-lab Model 1535, Sheldon Manufacturing Inc., Cornelius, OR, USA) for 72 h. Finally, the extract was recovered in solid form to prepare 2000 μg/mL of a stock solution for the antibacterial activity tests and the phytochemical composition analysis [15,19,34,35].

### 4.3. Phytochemical Extract Analysis

The ethanolic powdered extract of *M. tamaulipana* leaves was analyzed to conduct the phytochemical qualitative detection tests, including carbohydrates (Molisch’s test), reducing sugars (Fehling and Benedict’s tests), alkaloids (Dragendorff and Sonneschain’s tests), flavonoids (Shinoda’s test and NaOH test), cyanogenic glycosides (Gringnard’s test), saponins (foam test), sterols and terpenes (Liberman-Burchard’s test), tannins {FeCl_3_, K_3_[Fe(CN)_6_] and gelatine tests}, quinones (Börntraguer’s test), purins (HCl tests), polysaccharides (Lugol’s test), soluble starch (KOH and H_2_SO_4_ tests), and carotenoids (H_2_SO_4_ and FeCl_3_ tests) [36,37].

### 4.4. Experimental Design

We used the sand technique described by Ahmadi [38], with modification. Bean discs of 2.5 cm in diameter were cut and placed on water-soaked cotton, with the underside facing up. A disc was placed inside a 5 cm wide Petri dish; each disc had 10 adult ABM females with age of one day. The experiment was done under laboratory conditions at 27 ± 1 °C, 70–80% relative humidity (RH), and a photoperiod of 12:12 (light: darkness). The bean leaf discs were split at random into eight groups: a control group and seven treatment groups, one per each extract concentration. A bean leaf disc was the replicate. We had three replicates per group. Ten mite females were placed on each bean leaf disc and they were sprayed one time (0.5 ± 0.1 mL) with each concentration. We determined the spread amount of the extract with the help of a 2 mL sterile storage tube. We sprayed ten times into the storage tube to calculate the average and standard deviation of the amount sprayed. A manual sprayer (Truper^®^ Model 14687, Ciudad Victoria, Mexico) was used to apply *M. tamaulipana* extract at different concentrations (5, 10, 50, 100, 250, 500, 1000 µg/mL). The control treatment was sprayed with distilled water only.

### 4.5. Oligonychus punicae Oviposition

We counted the number of eggs at 24, 48, and 72 h with the help of a dissecting microscope (UNICO Stereo and Zoom Microscopes ZM180, Princeton, NJ, USA). The Kramer and Mulla [39] formula was used to determine the oviposition activity index (OAI) in each concentration.

OAI = [(NT − NC)/(NT + NC)](1)

where NC is the number of eggs in the control group and NT is the number of eggs in the treated group with the extract. The percentage values ranged between +1 and −1. The positive values indicate more eggs laid in the treatment than in the control group (showing that the extract stimulates egg-laying activity). In contrast, more eggs laid in the control group than in the treatment results in a negative OAI, indicating that the extract inhibits the egg-laying activity.

### 4.6. Oligonychus punicae Feeding

We registered the percentage of feeding damage at 24, 48, and 72 h with the help of a dissecting microscope (UNICO Stereo and Zoom Microscopes ZM180, Princenton, NJ, USA). The anti-feeding effect was measured through the inhibition percentage of feed intake by ABM in the bean leaf discs, as compared to the control treatment. At 24, 48, and 72 h, we classified the symptoms observed in the bean leaf discs according to an ordinal scale developed by Hussey and Parr [40] and Nachman and Zemek [41]. We converted this into percentages: 0 = 0% damage (with no feeding damage), 1 = 1–20%, 2 = 21–40%, 3 = 41–60%, 4 = 61–80%, and 5 = 81–100% of feeding damage (dense marks, or wilting, after eating all the bean disc). We used the criterion of Kramer and Mulla [39] formula to measure the females’ food intake. Positive values show that there is more damage in the treatment group than in the control group, indicating that the treatment promotes feeding. Negative values represent more severe damage to the control group than to the treatment group, indicating that feeding is inhibited in the treatment group.

### 4.7. Oligonychus punicae Growth Population

The rate of increase (r, day^−1^), the finite rate of increase (λ), and the population doubling time (D_T_) were used as the parameters to establish population increase of ABM, and r was calculated by [42]:

r = (1/t) × ln(Nt/N0)(2)

where Nt is the number of individuals at time t (surviving adult females plus the eggs laid at the end of the bioassay), N0 is the number of individuals at time 0 (initial cohort = 10 adult females of *O. punicae*), and t is the number of days elapsed from the start to the end of the bioassay (equal to 3 days). The finite growth rate, i.e., the number of times the population multiplied in a unit of time, was calculated as:

λ = antiloge r(3)

The doubling time was calculated by:

DT = Ln(2)/r(4)

The demographic parameters (r, λ, and DT) indicate the population growth of the red spider mite.

### 4.8. Statistical Analysis

The number of laid eggs, percentage of feeding damage, rate of increase (r, day^−1^), finite rate of increase, and doubling time were analyzed using analysis of variance (ANOVA) with mean comparison tests using Tukey’s HSD. We used a probit analysis to estimate the effective concentration [EC_50(90)_], including corresponding CI_95_ values, which caused oviposition inhibition by 50(90)% compared to the control [43]. Finally, we correlated the mean number of eggs laid per female with the average percent of feeding damage of *O. punicae*. The SAS/STAT software was used for all analyses [44].

## 5. Conclusions

The results show ethanolic powdered extract of *M. tamaulipana* leaves can be used in mite control. The extract reduced both feeding damage and oviposition of *O. punicae*, leading to lower growth rates. Further research is necessary, including the assessment of other types of *M. tamaulipana* extracts to control *O. punicae* and other species of pest mites, as well as studying the extract’s effect on natural enemies.

## Figures and Tables

**Table 1:** Secondary metabolites (“+” = present; “−” = absent) in Magnolia tamaulipana leaf ethanolic powdered extract.

Bioactive Compound		Test	Bioactive Compound		Test
Tannins	+	FeCl_3_ (catechol)	Flavonoids	+	Shinoda’s
	+	K_3_[Fe(CN)_6_]		+	NaOH
	+	Gelatine	Saponins	+	Foam
Carbohydrates	+	Molisch’s	Sterols and terpenes	+	Burchard’s
Carotenoids	+	H_2_SO_4_	Glycosides	−	Gringnard’s
	+	FeCl_3_	Quinones	+	Börntraguer’s
Coumarins	−	NH_4_OH		+	H_2_SO_4_
Free reducing sugars	+	Fehling	Soluble starch	+	KOH and H_2_SO_4_
Alkaloids	+	Benedict’s		+	Gelatine
	−	Dragendorff	Purins	+	HCI
	−	Sonneschain’s	Polysaccharides	−	Lugol’

**Table 2:** Effect of ethanolic powdered extract of *Magnolia tamulipana* leaves on oviposition of *Oligonychus punicae*.

Treatment	Eggs/Female ± SE *		
	24 h	48 h	72 h
Control	5.20 ± 0.51 a **	6.60 ± 0.12 a	6.87 ± 0.62 a
5 µg/mL	5.17 ± 0.18 a	5.07 ± 0.26 b	4.97 ± 0.12 ab
10 µg/mL	5.10 ± 0.23 a	4.23 ± 0.23 bc	3.97 ± 0.22 bc
50 µg/mL	4.43 ± 0.23 a	3.93 ± 0.22 cd	3.57 ± 0.88 bc
100 µg/mL	3.03 ± 0.15 b	3.07 ± 0.23 de	3.07 ± 0.18 cd
250 µg/mL	2.50 ± 0.12 bc	2.1 ± 0.31 ef	2.10 ± 0.21 de
500 µg/mL	1.53 ± 0.15 cd	1.37 ± 0.03 f	1.13 ± 0.17 e
1000 µg/mL	1.27 ± 0.18 d	1.17 ± 0.07f	0.80 ± 0.30 e
EC_50_ (IC_95_)	EC_90_ (IC_95_)	b ± EE	χ^2^
308.16	2982	2.23 ± 0.43	26.74 ***
(184.06–430.17)	(1718–9240)		

* Average number of eggs laid per female ± standard error (SE). ** Means (± SE) within a column and followed by different lowercase letters are significantly different (*p* ≤ 0.05; ANOVA and Tukey’s HSD test). EC: Effective concentration EC_50_ (EC_90_) in µg/mL causing 50% (90%) inhibition of egg laying by *Oligonychus punicae* females, compared with untreated control. CI: Confidence interval at 95%. b: slope ± standard error. χ^2^: Chi square value. *** Level of significance *p* < 0.0001.

**Table 3:** Effects of ethanolic powdered extract of *Magnolia tamaulipana* leaves on *Oligonychus punicae* feeding damage.

Treatment	Percentage of Feeding Damage ± SE *		
	24 h	48 h	72 h
Control	10.00 ± 0.58 a	17.33 ± 1.45 a	29.00 ± 1.00 a
5 µg/mL	8.67 ± 0.33 ab	14.67 ± 0.33 ab	21.00 ± 1.00 b
10 µg/mL	8.67 ± 0.88 ab	14.67 ± 1.86 ab	20.00 ± 0.58 b
50 µg/mL	8.00 ± 0.58 abc	12.00 ± 1.53 bc	19.67 ± 1.76 b
100 µg/mL	7.00 ± 0.58 abc	8.67 ± 0.33 cd	15.33 ± 1.45 bc
250 µg/mL	6.33 ± 0.67 bc	7.00 ± 1.00 cd	12.67 ± 1.15 cd
500 µg/mL	5.00 ± 0.58 cd	6.67 ± 0.33 d	8.67 ± 0.88 de
1000 µg/mL	2.67 ± 0.88 d	4.33 ± 0.33 d	5.67 ± 0.88 e

* Means ± standard error (SE) within a column and followed by different lowercase letters are significantly different (*p* ≤ 0.05; ANOVA and Tukey’s HSD test).

**Table 4:** Effects of *Magnolia tamaulipana* leaf ethanolic powdered extract on the rate of increase, the finite rate of increase, and the population doubling time of *Oligonychus punicae*.

Treatment	Demographic Parameters		
	Growth Rate *	Finite Rate of Growth	Doubling Time
Control	0.9929 ± 0.01 a	2.6991 ± 0.01 a	0.6982 ± 0.00 f
5 µg/mL	0.9283 ± 0.00 b	2.5303 ± 0.01 b	0.7467 ± 0.00 ef
10 µg/mL	0.8865 ± 0.01 bc	2.4268 ± 0.02 c	0.7821 ± 0.01 ef
50 µg/mL	0.8532 ± 0.00 c	2.3473 ± 0.01 c	0.8124 ± 0.00 de
100 µg/mL	0.7729 ± 0.01 d	2.1662 ± 0.01 d	0.8969 ± 0.01 d
250 µg/mL	0.6796 ± 0.02 e	1.9736 ± 0.03 e	1.0212 ± 0.03 c
500 µg/mL	0.5376 ± 0.02 f	1.7125 ± 0.03 f	1.2929 ± 0.05 b
1000 µg/mL	0.4809 ± 0.01 g	1.6175 ± 0.01 f	1.4421 ± 0.02 a

* Means ± standard error (SE) within a column and followed by different lowercase letters are significantly different (*p* ≤ 0.05; ANOVA and Tukey’s HSD test).

## Data Availability

The data presented in this study are available on request from the corresponding author.
